# Supplementary material for: The role of survivin in the progression of pancreatic ductal adenocarcinoma (PDAC) and a novel survivin-targeted therapeutic for PDAC
Source: PLoS One. 2020 Jan 13;15(1):e0226917. doi: 10.1371/journal.pone.0226917 (PMC6957139; doi:10.1371/journal.pone.0226917)
Supplement: S1 File — Included in this file are the original, uncropped images of the western blot membranes taken at the time of exposure. In the raw images, the membranes were cut in order to probe for multiple proteins on the same membrane at the same time. An image of the protein ladder (from Bio-Rad) is included next to the raw blot image for reference. Each page of the file represents a single exposure. (PDF) [file pone.0226917.s001.pdf]

48-hr Treatment Blot

Blot developed using chemiluminescence UV exposure with BioRad ChemiDoc Systems

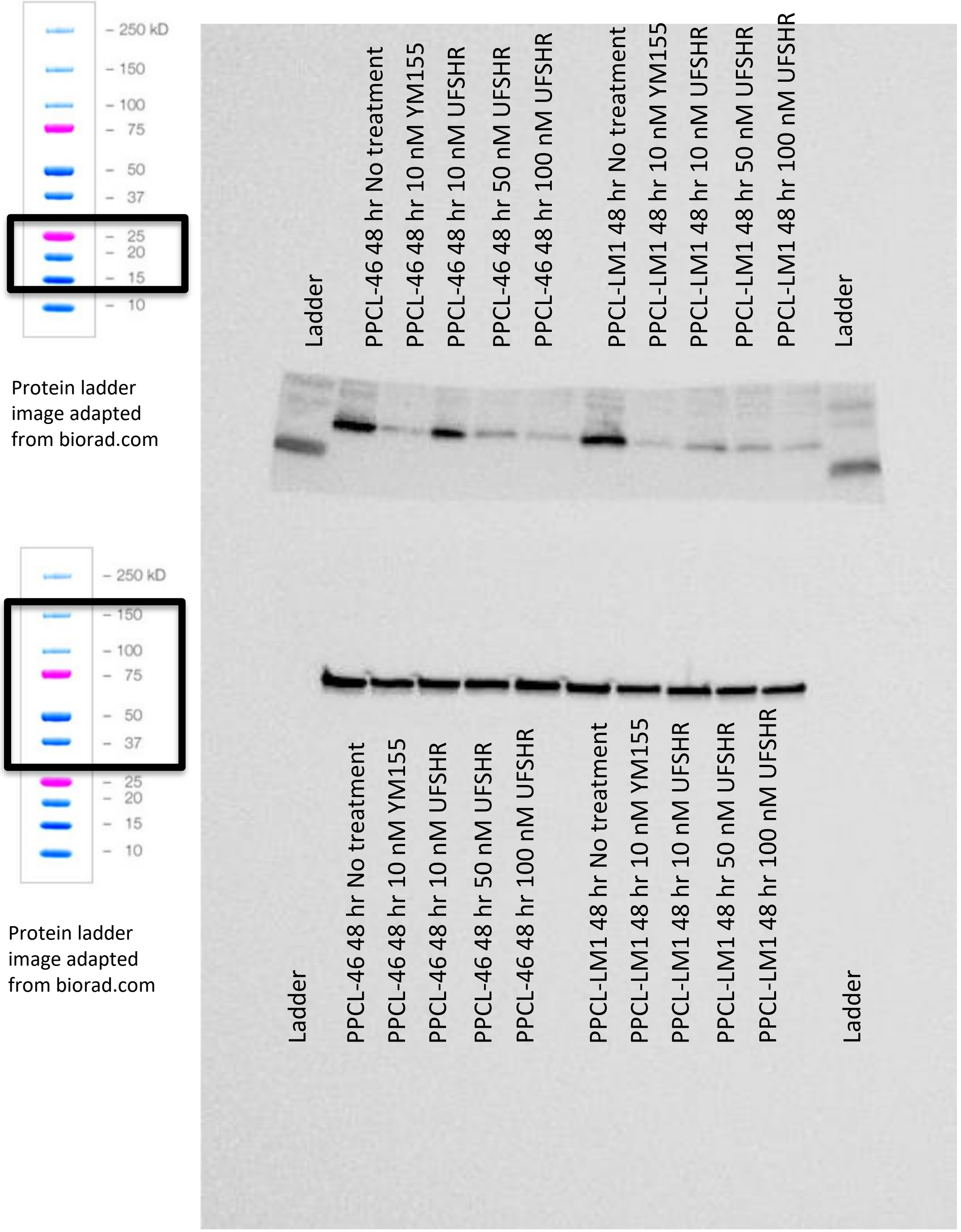

Protein of Interest: Survivin

Protein of Interest: Actin

Figure 2B

Blot developed using chemiluminescence UV exposure with BioRad ChemiDoc Systems

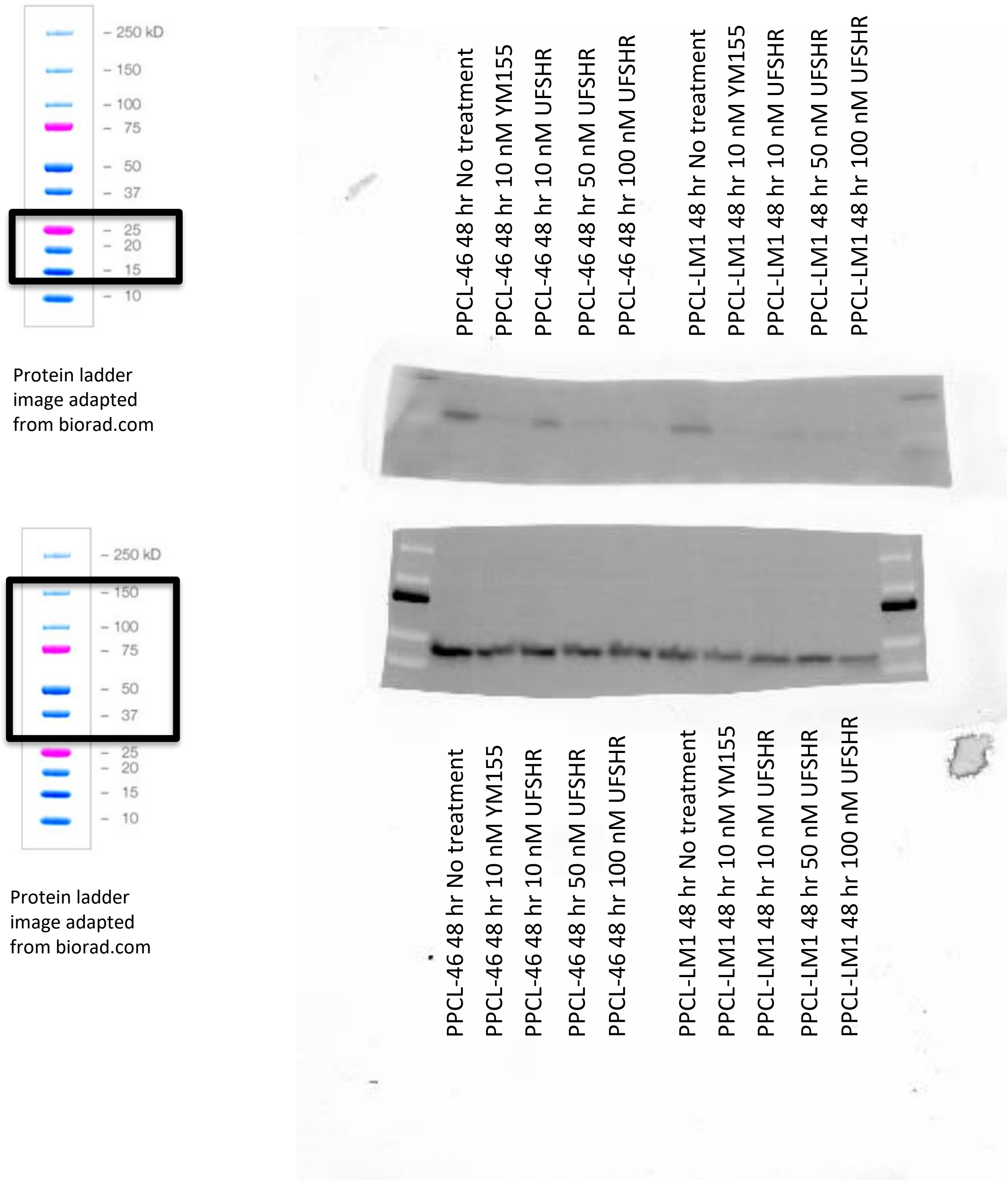

Protein of Interest: Survivin

Protein of Interest: Actin

Figure 2B

24-hr Treatment Blot

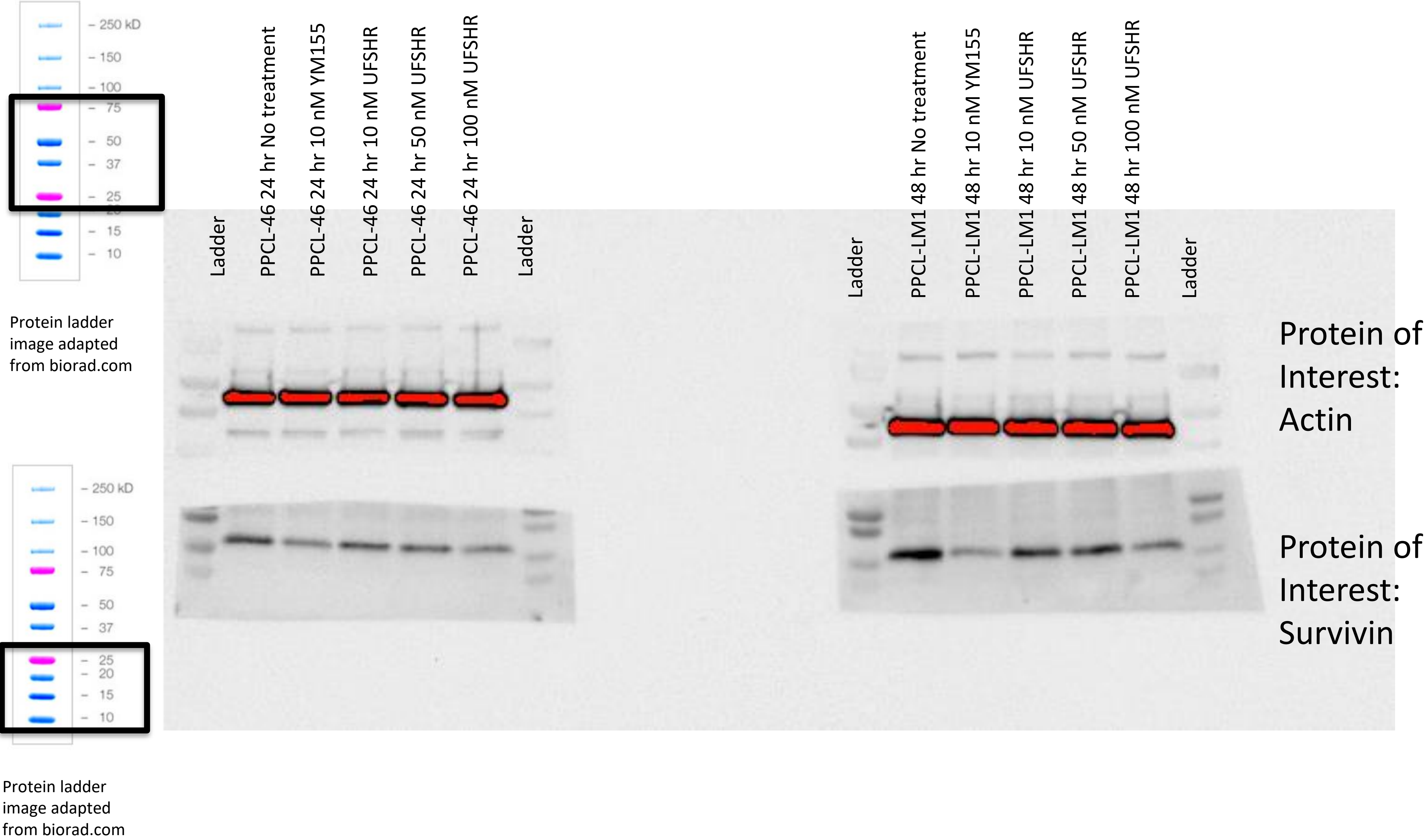

Blot developed using chemiluminescence UV exposure with BioRad ChemiDoc Systems

Figure 2B

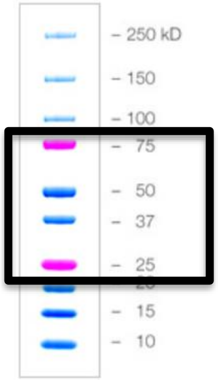

Protein ladder image adapted from biorad.com

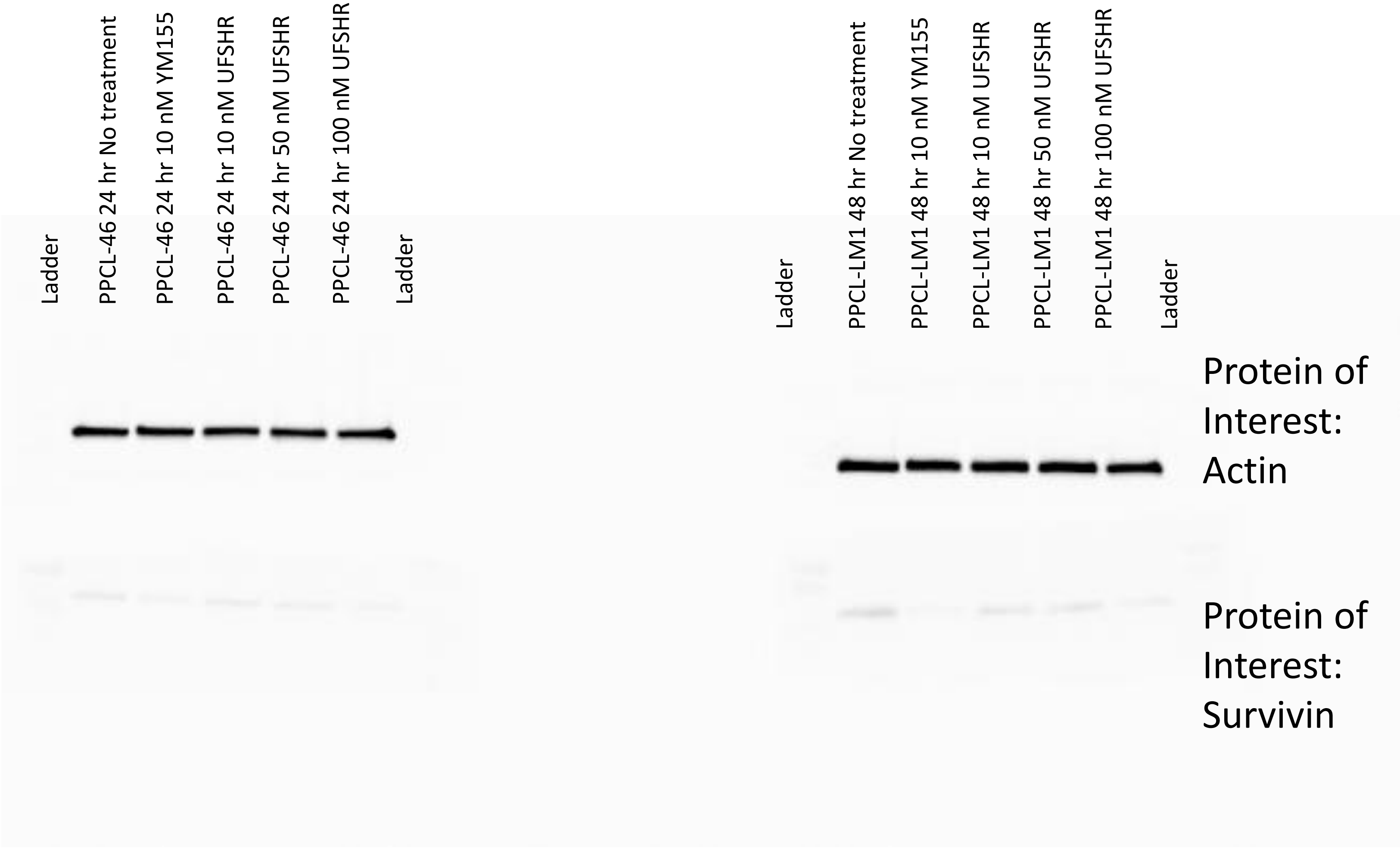

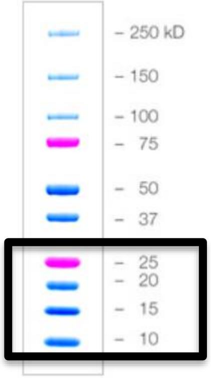

Protein ladder image adapted from biorad.com

Blot developed using chemiluminescence UV exposure with BioRad ChemiDoc Systems

Figure 2B
