# Supplementary material for: The role of survivin in the progression of pancreatic ductal adenocarcinoma (PDAC) and a novel survivin-targeted therapeutic for PDAC
Source: PLoS One. 2020 Jan 13;15(1):e0226917. doi: 10.1371/journal.pone.0226917 (PMC6957139; doi:10.1371/journal.pone.0226917)
Supplement: Attachment — Submitted filename: Response to Reviewers.pdf [file pone.0226917.s002.pdf]

**Manuscript: PONE-D-19-19723**

**Title:** ***The Role of Survivin in the Progression of Pancreatic Ductal Adenocarcinoma (PDAC) and a Novel Survivin-Targeted Therapeutic for PDAC***

We would like to thank the reviewers for their time and their effort to evaluate this manuscript. We have made some major and minor changes throughout the manuscript, according to the reviewers' comments. Our point-to-point responses to specific criticisms were addressed below.

Reviewer's comments:

Reviewer #1: The authors present data validating survivin as a target in pancreatic adenocarcinoma using TCGA data and histologic evaluation of primary tissues compared to histologic normal tissue. There have been earlier publications showing survivin is upregulated in pancreatic cancer (Satoh. Cancer 2001;92:271–278) and its inhibition using knockdown sensitizes pancreatic CA to radiation (Kami. Surgery 2005;138:299–305).

**The upregulation of survivin in pancreatic cancer (PC) has been reported in some studies, which is consistent with our data presented in this manuscript using patient-derived xenograft PC models. Despite this interesting observation, the therapeutic potential of survivin is underestimated in pancreatic cancer, both in pre-clinical and clinical settings:**

**In preclinical studies using highly passaged PC cell lines, other groups have showed that inhibition of survivin either by siRNA or YM155, a highly potent inhibitor of survivin, can re-sensitize PC cells to radiation or to gemcitabine, respectively (Kami et.al., Surgery 2005;138:299–305 and Yoon et.al., Anticancer Res 2012, 32:1681-1688). We did attempt to evaluate the combination effect of survivin inhibitors (YM155 and UFSHR) and gemcitabine. However, no significant improvement in efficacy of combination treatment was observed in our PDX PC lines (data not shown in the manuscript).**

**In the clinical setting, YM155, a highly potent inhibitor of survivin, is undergoing Phase I and Phase II clinical trials for different types of solid tumors including melanoma, non-small cell lung cancer, breast, and prostate cancers, etc, but not pancreatic cancer. The only trial targeting survivin for pancreatic cancer was recently reported in the publication: Shima et.al., Cancer Sci. 2019, 110(8):2378-2385. This randomized phase II trial of survivin 2B peptide (SVN-2B) vaccination for patients with HLA-A24-positive pancreatic cancer showed that patients vaccinated with SVN-2B plus IFNβ did not have improved PFS, but showed significant immunological reaction after vaccination. This study also suggested that a longer SVN-2B plus IFNβ vaccination protocol might confer survival benefit.**

**Given the urgent need for better treatment of highly lethal pancreatic cancer, we aimed to reinforce the great therapeutic potential for targeting survivin in this cancer through the evaluation of a novel survivin inhibitor in more reliable and clinically relevant PDX PC models. This is the main purpose of this manuscript.**

The authors use unique patient derived cell line models-one is from the primary cancer and other is derived from a metastatic pancreatic cancer lesion. It would be interesting to see at what stage in transformation this protein is upregulated in pancreatic adenoCA, looking at the continuum of adenomas, in situ and invasive carcinoma-primary or metastatic lesions. Moreover with these 2 different cell lines differences seen in the efficacy of the survivin inhibition may be related to differences in the relevance of this protein in primary versus metastatic lesion.

**We highly agree with the reviewer on this point. In fact, we have looked into the correlation/transition of survivin expression during the stages of disease progression, including hepatic metastasis. Unfortunately, we failed to provide a statistically valid conclusion, due to insufficient number of studied cases in each category. In another attempt, we analyzed the distribution of mRNA level (measured by FPKM, Fragments Per Kilobase of transcript per Million mapped reads) in 176 samples of PC patients with different stages (TCGA database). We did not find any statistical evidence for the correlation of survivin expression and disease stages, due to a lack of samples in studied groups (attached graph).**

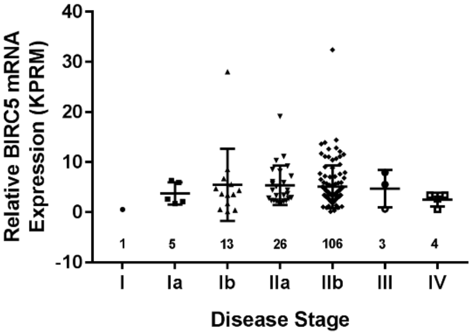


Another novelty of this research is the use of UFSHR a novel derivative of YM155 which is a known survivin inhibitor by destabilizing the transcriptional complex at the BIRC promoter. However, the authors have not confirmed the mechanism by which this drug is inhibiting survivin – there is no examination of survivin gene expression to confirm transcriptional repression of BIRC5. Are there differences in BIRC8 which is upregulated by YM155 as an off target effect? Do the 2 drugs differ in their effects on survivin splice variants that are responsible for the anti-apoptotic effects of this gene.

**We agree with the reviewer that understanding the mechanism of this novel survivin inhibitor UFSHR is crucial, however, it is not within the scope of this manuscript. Herein, we focused the first on the functional impact of this new inhibitor on survivin-regulated tumorigenicity of pancreatic cancer. The very encouraging data reported for the first time in this study provide us a solid evidence for therapeutic potential of UFSHR, from which we will move forward to investigate its mechanism of action. In the next step, we will continue to explore not only the survivin-inhibiting mechanism of UFSHR (particularly its role in survivin's mRNA/protein regulation, its specificity compared to YM155, the inhibitory efficacy on survivin splice variants, etc.) but also the difference/similarity in the inhibitory effect of UFSHR, compared to that of YM155, on survivin-regulated signaling cascades in pancreatic cancer. Currently, this proteomic analysis is being conducting with a powerful, high-throughput, and quantitative protein array platform. Therefore, we plan to analyze the impact of UFSHR and YM155 on more than 100 cancer-related signaling pathways in PDX derived PC cell lines.**

**The mechanism of YM155-induced survivin repression has been cited in the past as principally acting through transcriptional inhibition. However, while YM155 does in fact reduce survivin mRNA levels and this likely contributes to the reduction seen in survivin protein levels, more recent studies have shown that the mechanism whereby YM155 downregulates survivin protein expression seems to be primarily due to changes in complex translational control cascades. Polysome profiles were generated in renal and prostate cancer cell lines following YM155 treatment which showed that YM155 substantially suppresses cap-dependent translation of mRNAs that include survivin (Danielpuor et. al., Nature Scientific Reports, 2019). Before any noticeable changes in mRNA transcripts, this experiment showed a significant suppression in translation as early as 4 hours after the start of YM155 treatment. This study also showed that YM155 can change the phosphorylation status of known mTOR-target proteins that regulate translation (i.e. ribosomal protein S6 (rS6) and 4E-BP1). The last critical evidence from this study revealed that when cells were treated with**

**MG132 (a proteasome inhibitor), there was no significant reversal of survivin loss by YM155, indicating that YM155 likely does not act through a proteasomal mechanism.**

**Consistent with this recently published data describing the translational control of survivin expression, our own RT-PCR experiments showed that the YM155-analog UFHSR had a negligible impact on mRNA expression. However, UFSHR showed a potent effect on survivin protein expression in immunoblotting studies. It is possible that this YM155 analog UFSHR acts either through one of the translational control mechanisms targeted by YM155 or an entirely different mechanism of translational inhibition of survivin due to the changes in critical chemical functional groups. Therefore, this supports our future plan to conduct a comprehensive proteomic analysis in order to fully capture the mechanism of action of UFSHR on the translational control of survivin and to ascertain how this impacts downstream tumor cell signaling. Meanwhile, UFHSR's effect on survivin-regulated phenotypes *in vitro* and its significant reduction of tumor progression *in vivo* strongly supports the continued evaluation of UFHSR as a novel survivin-targeted therapeutic for PDAC.**

The concept of inhibiting survivin in this cancer is not novel- a peptide survivin vaccine in combination with interferon in a phase 1 trial in pancreatic cancer was published in Cancer Science in 2018. The PDX animal data is compelling. This drug is inhibiting tumor growth and suppressing the target in vivo. Whether survivin is critical to this anticancer activity of UFSHR should be proven by overexpressing survivin to reverse the apoptotic phenotype because destabilizing a transcriptional complex could have multiple effects.

**We agree that the concept of inhibiting survivin in this cancer is not novel. The randomized phase II trial mentioned by the reviewer so far is the only one. Given the fact 1) there is a limited number of clinical trials targeting survivin in PC and 2) that the 5-year survival rate is still extremely low in patients diagnosed with PC, we think there is an urgent need for identifying new therapeutic options to effectively treat this aggressive and dismal disease. The novelty of this research is UFSHR in its role as a new survivin inhibitor that has the potential to become a small molecule therapy for the treatment of PC. In addition, as the reviewer has pointed out, a crucial element to our preclinical evaluation of UFSHR has been the reliable and clinically relevant PDX models. This is an essential step to move forward to developing novel treatment approaches in precision medicine. Indeed, our data demonstrated a significant difference in sensitivity of each drug between 2 different models. UFSHR showed a significant inhibitory effect on tumor growth in both PPCL-46 and PPCL-LM1 models, while YM155 is only sensitive in the PPCL-LM1 tumor.**

**The reviewer's suggestion on testing the activity of UFSHR on survivin-overexpressing cells is a great idea to validate the specificity of this inhibitor to survivin. In fact, we are establishing a survivin-overexpressing HPDE cell line with CRISPR/Cas9. This human pancreatic duct epithelial cell line has very little to no survivin expression (Figure 1D). The role of survivin in cancer transformation and the anti-cancer effect/specificity of UFSHR will be tested afterward. This experiment will be included in the next phase of our study that which will focus on the underlying mechanism of UFSHR.**

Statistics have not been shown to justify the size of the animal experiments nor is there any mention of any statistical tests used in the in vitro experiments.

**We have incorporated statistical tests into both *in vitro* and *in vivo* studies, specifically as listed below:**

**Figure 2: we added new data on mRNA expression with statistical analysis included.**

**Figure 3: statistical analysis was represented in the new bar graph, highlighting the significant difference in viable and apoptotic populations in the presence/absence of YM155 or UFSHR.**

**Figure 4: statistical analysis was represented in the new bar graph, showing the significant change in the unoccupied area of cancer cells in the presence/absence of YM155 or UFSHR.**

**Figure 5: we added new quantitative data on survivin expression in tumors with statistical analysis included.**

**Materials and Methods: statistical analysis was added to each procedure as well as the statistical analysis section.**

**Legends: symbols representing statistical significance were clarified in this section.**

Journal Requirements:

1. Please ensure that your manuscript meets PLOS ONE's style requirements, including those for file naming. The PLOS ONE style templates can be found at http://www.journals.plos.org/plosone/s/file?id=wjVg/PLOSOne_formatting_sample_main_body.pdf and http://www.journals.plos.org/plosone/s/file?id=ba62/PLOSOne_formatting_sample_title_authors_affiliations.pdf

**We have reformatted our original manuscript to meet all formatting and style requirements.**

2. In your Methods section, please provide additional information about the pancreatic tumor samples used to derive the PDX models. Please ensure you have provided sufficient details to replicate the analyses such as: a) the date range (month and year) during which patient samples were collected, b) a description of relevant demographic details (sex, stage, molecular subtype) and c) the number of independent samples used in this work.

**This information has been updated in the Materials and Methods section.**

3. PLOS ONE now requires that authors provide the original uncropped and unadjusted images underlying all blot or gel results reported in a submission's figures or Supporting Information files. This policy and the journal's other requirements for blot/gel reporting and figure preparation are described in detail at https://journals.plos.org/plosone/s/figures#loc-blot-and-gel-reporting-requirements and https://journals.plos.org/plosone/s/figures#loc-preparing-figures-from-image-files. When you submit your revised manuscript, please ensure that your figures adhere fully to these guidelines and provide the original underlying images for all blot or gel data reported in your submission. See the following link for instructions on providing the original image data: https://journals.plos.org/plosone/s/figures#loc-original-images-for-blots-and-gels.

In your cover letter, please note whether your blot/gel image data are in Supporting Information or posted at a public data repository, provide the repository URL if relevant, and provide specific

details as to which raw blot/gel images, if any, are not available. Email us at plosone@plos.org if you have any questions.

**In the file entitled "F2B_raw_images.pdf", we have included the original, uncropped images of the western blot gels taken at the time of exposure in accordance with the guidelines provided on the PLOS ONE website. In the raw images, the membranes were cut in order to probe for multiple proteins on the same membrane at the same time. Each page represents a single exposure.**
